# Editorial: Mineral and Bone Disorder in CKD

**DOI:** 10.3389/fped.2022.856656

**Published:** 2022-02-18

**Authors:** Nikoleta Printza, John Dotis, Manish D. Sinha, Maren Leifheit-Nestler

**Affiliations:** ^1^Pediatric Nephrology Unit, 1st Pediatric Department, Hippokration General Hospital, Aristotle University, Thessaloniki, Greece; ^2^Department of Paediatric Nephrology, King's College London, Evelina London Childrens Hospital, London, United Kingdom; ^3^Department of Pediatric Kidney, Liver and Metabolic Diseases, Pediatric Research Center, Hannover Medical School, Hanover, Germany

**Keywords:** mineral and bone disorder, chronic kidney disease, children, secondary hyperthyroidism, renal transplantation

Mineral and bone disorder (MBD) is defined as a systemic abnormality of mineral and bone metabolism, which usually accompanies chronic kidney disease (CKD), with subsequent deleterious effects in bone histomorphometry, skeletal growth and strength as well as vascular tissue of children with CKD (1). In this Research Topic, we provide the reader with a comprehensive and current update on various aspects of the disease, including recent epidemiological data of MBD clinical manifestations, recommended strategies and novel tools for optimization of MBD assessment and treatment, emerging evidence regarding the impact of the disease on cardiovascular and other systems and detection of novel pathogenetic pathways. A prominent group of 31 authors have contributed to this issue with a total of 6 articles, including 3 review papers and 3 clinical studies.

Growth retardation constitutes a major complication, which impacts on life quality and aggravates overall kidney disease associated morbidity. Although significant catch-up growth occurs following kidney transplantation (KTx), and is more evident in the youngest patients, growth delay unfortunately may persist (2). Therefore, systematic and accurate monitoring of height velocity is crucial for early diagnosis of growth failure, and prompt therapeutic interventions essential for preventing short stature. We introduce this Research Topic with a mini review, written by Haffner, summarizing the cornerstones of currently recommended clinical measures to optimize growth in children with CKD.

Lopez-Gonzalez et al. extend these practice points and present data from a retrospective single-center study, informing the incidence and risk factors of growth retardation following KTx. More than two thirds of children finally achieved their expected adult height, and this was primarily attributed to the efficient use of growth hormone (rGH) before and following KTx. These findings support the safety and efficacy of rGH therapy as an adjuvant treatment for growth delay in pediatric kidney transplant recipients. As expected, steroid therapy was negatively associated with final height, highlighting the importance for minimization of steroid exposure in pediatric transplant recipients. Finally, height SDS (standard deviation score) at KTx, which was lower in the youngest patients, was the principal association of subsequent growth, raising the special interest in targeted preventive and therapeutic strategies at this vulnerable population.

The currently recommended serum biomarkers for monitoring pediatric CKD-MBD include calcium, phosphorus, alkaline phosphatase, parathormone (PTH) and 25-hydroxy vitamin D levels (3). The role of other biomarkers for optimal assessment of bone mineral MBD in children with CKD has gained increasing research interest. Toward this direction, in this Research Topic, Schmitz et al. studied the impact of enteral calcium intake (Ca-I) on the evaluation of mineral balance in children aged between 0 and 6 years old with CKD. In this retrospective multicenter analysis, children with CKD were prone to both under and overexposure to enteral calcium intake, indicating the need for Ca-I systematic monitoring. Interestingly, enteral Ca-I was significantly negatively correlated to serum PTH, despite no correlation to serum calcium or serum phosphorus, suggesting that enteral Ca-I may serve as a valuable contributive marker of calcium balance and a promising target for more efficient management of secondary hyperparathyroidism.

Since the discovery of fibroblast growth factor 23 (FGF23) in 2000, several investigational studies have been focused on the contribution of this bone-derived hormone in the manifestation of CKD-MBD, and its possible impact on the enhancement of cardiovascular disease (4, 5). In this Research Topic, Grund et al. present a state-of the art review paper regarding the cardiotoxic effects of FGF23 and its contributive role in the development of left ventricular hypertrophy (LVH) in CKD, based on *in-vitro, in-vivo* and clinical studies. Considering that FGF23 may be not only a biomarker but also a driver of LVH, the authors additionally analyze the emerging strategies targeting the FGF23-αKlotho-axis in order to ameliorate uremic cardiomyopathy. They additionally highlight the relative paucity of clinical data in this area for children with CKD.

In this Research Topic, Karava et al. provide new insights in the pathogenesis of CKD-MBD. The authors analyze the adipose tissue endocrine effects on mineral balance from experimental and clinical studies and highlight the clinical data supporting a contributive role of obesity on the development of CKD-MBD. In parallel, they describe in detail the contrary modulatory effect of mineral status on adipose tissue metabolism and underline the emerging clinical evidence regarding the role of disturbed mineral balance on CKD related fat loss.

Further, Karava et al. present the results of a cross-sectional study on the association of secondary hyperparathyroidism with body composition indices in pediatric patients CKD. In this study, serum PTH was positively associated with adiposity in moderate but not in advanced CKD, suggesting that in children with CKD and high adiposity may present secondary hyperparathyroidism earlier in the course of the disease. Moreover, the authors observed a negative association between alfacalcidol index, defined in their study as weekly alfacalcidol dose per PTH level, and muscle wasting in advanced CKD, suggesting that alfacalcidol index may also serve as a marker of nutritional status. Insights from these data regarding the contributive effect of high PTH on muscle wasting and the protective role of active vitamin D supplementation on muscle preservation suggest further studies are needed, to explore the pathogenic mechanisms linking muscle status and mineral balance in CKD with subsequent clinical outcomes (6).

Although much has been achieved, management of CKD-MBD remains a clinical challenge for pediatric nephrologist. Further research is required, including large-scale observational studies and well-conducted randomized controlled trials, to evaluate the appropriate therapeutic interventions that will optimize bone health in children with CKD. Moreover, large scales cohort studies are crucial to enlighten the early recognition of the potential adverse effects of CKD-MBD on the other systems, in order to reduce overall morbidity of pediatric patients. Finally, investigations regarding novel pathogenic pathways of CKD-MBD and advanced clinical tools targeting better comprehension of patient mineral status may improve our knowledge toward a more holistic approach of this disorder.

## Author Contributions

All authors listed have made a substantial, direct, and intellectual contribution to the work and approved it for publication.

## Conflict of Interest

The authors declare that the research was conducted in the absence of any commercial or financial relationships that could be construed as a potential conflict of interest.

## Publisher's Note

All claims expressed in this article are solely those of the authors and do not necessarily represent those of their affiliated organizations, or those of the publisher, the editors and the reviewers. Any product that may be evaluated in this article, or claim that may be made by its manufacturer, is not guaranteed or endorsed by the publisher.
